# A flexible R package for nonnegative matrix factorization

**DOI:** 10.1186/1471-2105-11-367

**Published:** 2010-07-02

**Authors:** Renaud Gaujoux, Cathal Seoighe

**Affiliations:** 1 Computational Biology Group, Department of Clinical Laboratory Sciences, Faculty of Health Sciences, University of Cape Town, South Africa; 2 School of Mathematics, Statistics and Applied Mathematics, National University of Ireland Galway, Ireland

## Abstract

**Background:**

Nonnegative Matrix Factorization (NMF) is an unsupervised learning technique that has been applied successfully in several fields, including signal processing, face recognition and text mining. Recent applications of NMF in bioinformatics have demonstrated its ability to extract meaningful information from high-dimensional data such as gene expression microarrays. Developments in NMF theory and applications have resulted in a variety of algorithms and methods. However, most NMF implementations have been on commercial platforms, while those that are freely available typically require programming skills. This limits their use by the wider research community.

**Results:**

Our objective is to provide the bioinformatics community with an open-source, easy-to-use and unified interface to standard NMF algorithms, as well as with a simple framework to help implement and test new NMF methods. For that purpose, we have developed a package for the R/BioConductor platform. The package ports public code to R, and is structured to enable users to easily modify and/or add algorithms. It includes a number of published NMF algorithms and initialization methods and facilitates the combination of these to produce new NMF strategies. Commonly used benchmark data and visualization methods are provided to help in the comparison and interpretation of the results.

**Conclusions:**

The NMF package helps realize the potential of Nonnegative Matrix Factorization, especially in bioinformatics, providing easy access to methods that have already yielded new insights in many applications. Documentation, source code and sample data are available from CRAN.

## Background

### Non-negative Matrix Factorization

The factorization of matrices representing complex multidimensional datasets is the basis of several commonly applied techniques for pattern recognition and unsupervised clustering. Similarly to principal components analysis (PCA) or independent component analysis (ICA), the objective of non-negative matrix factorization (NMF) is to explain the observed data using a limited number of basis components, which when combined together approximate the original data as accurately as possible. The distinguishing features of NMF are that both the matrix representing the basis components as well as the matrix of mixture coefficients are constrained to have non-negative entries, and that no orthogonality or independence constraints are imposed on the basis components. This leads to a simple and intuitive interpretation of the factors in NMF, and allows the basis components to overlap.

### Applications and motivations

Since its formal definition in [1,2], the NMF approach has been applied successfully in several fields including image and pattern recognition, signal processing and text mining [3]. NMF has also been applied to obtain new insights into cancer type discovery based on gene expression microarrays [4], for the functional characterization of genes [5], to predict cis-regulating elements from positional word count matrices [6] and, more recently, for phenotype prediction using cross-platform microarray data [7]. For a comprehensive review of applications of NMF in computational molecular biology see [3].

The popularity of the NMF approach derives essentially from three properties that distinguish it from standard decomposition techniques.

Firstly, the matrix factors are by definition nonnegative, which allows their intuitive interpretation as real underlying components within the context defined by the original data. The basis components can be directly interpreted as parts or basis samples, present in different proportions in each observed sample. In the context of gene expression microarrays, Brunet et al. [4] interpreted them as metagenes that capture gene expression patterns specific to different groups of samples. When decomposing positional word count matrices of k-mers in DNA sequences, Hutchins et al. [6] interpreted the basis samples as specific sequence patterns or putative regulatory motifs.

Secondly, NMF generally produces sparse results, which means that the basis and/or mixture coefficients have only a few non-zero entries. This provides a more compact and local representation, emphasizing even more the parts-based decomposition of the data [2]. NMF based representations has been shown to perform very well in the identification of clusters of samples and their characterization with a small set of marker features [3]. For example, Carmona-Saez et al. [8] used this property to define a bi-clustering approach for gene expression microarrays. Samples are first clustered together based on the metagene that most contributes to their expression profile. Then each cluster is characterized by the genes that specifically contribute the most to each metagene. The sparseness of the results is such that each set of metagene-specific genes remains limited, which facilitates the interpretation even more.

Finally, unlike other decomposition methods such as SVD or ICA, NMF does not aim at finding components that are orthogonal or independent, but instead allows them to overlap. This unique feature is particularly interesting in the context of gene expression microarrays, where overlapping metagenes could identify genes that belong to multiple pathways or processes [4,9].

### Formal definition

In this section we provide a mathematical formulation of the general NMF approach. Let *r *> 0 be an integer, and *X *a matrix with *n *rows - the measured features - and *p *columns - the samples - with only non-negative entries. Non-negative Matrix Factorization consists in finding an approximation(1)

where *W*, *H *are *n *× *r *and *r *× *p *non-negative matrices, respectively. Since the objective is usually to reduce the dimension of the original data, the factorization rank *r *is in practice often chosen such that *r *≪ min(*n, p*).

Equation (1) states that each column of *X *(i.e. the observed features of each sample) is approximated by a non-negative linear combination of the columns of *W *(i.e. the basis components), where the coefficients are given by the corresponding column of *H *(i.e. the mixture coefficients).

The main approach to NMF estimates matrices *W *and *H *as a local minimum of the following optimization problem:(2)

where *D *is a loss function that measures the quality of the approximation. Common loss functions are based on either the Frobenius norm or the Kullback-Leibler divergence [10,11]. *R *is an optional regularization function, defined to enforce desirable properties on matrices *W *and *H*, such as smoothness or sparsity [11]. Some variations on equation (1) and the optimization problem (2) are possible, depending on the application field or the a priori knowledge one has of the observed data [12,13].

### Existing implementations

Several algorithms to perform NMF have been published and implemented. See [14] for a review of some existing methods. When available, their implementations are usually in the form of MATLAB^® ^files. Hoyer provided a package that implements five different algorithms [12]. Cichocki and Zdunek produced the appealing NMFLAB package that implements a wide range of NMF algorithms, which can be combined, tested and compared via a graphical interface [15]. However, availability only in MATLAB^®^, a proprietary software, limits access to these packages within the wider bioinformatics community. Some C/C++ implementations are also available [16,17], including a parallel implementation using the Message Passing Interface (MPI) [18]. Finally a small number of R implementations exist [19,20], which are limited to specific NMF methods.

To help realize the potential of NMF, especially in bioinformatics, we have implemented a free open-source package, that allows users to use and implement NMF algorithms.

## Implementation

We implemented our package using the R/BioConductor platform [21,22], which is a well established and extensively used standard in statistical and bioinformatics research. We further motivate our choice by the fact that it is completely open-source and available for a wide range of operating systems; it offers an easy integration and distribution of third-party packages; and last but not least, it benefits from a very active support community. Moreover, performing NMF on large scale data requires intensive computations; the ability of R to call external complied code (C/C++, Fortran) allows the integration of optimized implementations of the algorithms.

### Implemented methods

#### Algorithms

Six published algorithms are implemented, either directly or by porting available code to R. We ported to R the standard NMF algorithms with multiplicative updates of [10] and [4], as well as the Alternate Least Square (ALS) approach of [23]. Non-smooth NMF (nsNMF) from [13], NMF with offset from [24], and PE-NMF [25] were directly implemented, as these are modifications of the standard algorithms. Whenever possible, we carried out validation tests of our implementations on sample data, confirming that the results match those obtained using the algorithms' original implementations.

#### Seeding methods

The general NMF procedure is to run the algorithm with several random initializations for matrices *W *and *H*, and keep the factorization that achieves the lowest approximation error across the multiple runs. However, more sophisticated and deterministic initialization methods have been proposed to choose appropriate initial values to seed NMF algorithms [26,27]. The whole procedure then becomes deterministic and only needs to run once. The NMF package currently implements the classical random initialization, the non-negative double singular value decomposition (NNSVD) approach from [27], as well as an initialization based on Independent Component Analysis (ICA), where the entries for *W *and *H *are set to the positive part of the result from an ICA. Moreover, reproducible tests can be performed by specifying either a numerical seed for the random number generator, or an explicit factorization to be used as a starting point. This is useful when developing new algorithms or comparing the performance of different methods. Each seeding method can be combined with any of the implemented algorithms.

#### Stopping criteria

Although differing in the way the solution is updated at each iteration, some of the implemented algorithms share a common iterative schema, with common stopping criteria. The NMF package implements three standard criteria: fixed number of iterations, invariance of the consensus matrix [4], and stationarity of the objective value.

### Flexibility and extensibility

While implementing all the possible NMF algorithms is beyond the scope of this work, one of the main objectives of our package is to provide a flexible framework for using and developing NMF algorithms in R. Our implementation is based on the *strategy design-pattern *which enables the user to easily integrate new methods. Because both built-in and custom methods implement a common programmatic interface, they benefit from the same collection of utility functions to visualize and compare their results. Finally, we defined our framework in a very generic way so that it is not limited to any application field. However, we provide a layer that is specific to bioinformatics, based on the BioConductor platform, which facilitates the analysis and interpretation of biological data.

## Results

To illustrate the capabilities of the NMF package, we provide an example of analysis on a real dataset. We used the Golub dataset as referenced in Brunet et al. [4]. It contains Affymetrix Hgu6800 microarray data from 27 patients with acute lymphoblastic leukemia (ALL) and 11 patients with acute myeloid leukemia (AML). The ALL group is subdivided into the B and T subtypes, composed of 19 and 8 patients respectively. Only the 5,000 most highly varying genes according to their coefficient of variation were used. All the results shown in the following come from the application of NMF algorithms to this dataset, using the implementation available in our package. The computations were performed in R version 2.10.1, on a Dell PC Intel Core2 vPro 2 × 2.33 GHz, with 3.7 Go RAM, under Linux Ubuntu 9.04. Our results are similar to those presented in [4], with minor differences that can be attributed to differences in random number seeds.

### Running NMF algorithms

Particular care was taken to provide the user with a lean and intuitive programmatic interface. We organized our package around a *single interface *function, that reduces to the minimum the amount of code needed to perform common analysis. It is straightforward to run any implemented NMF algorithm, combine algorithms and seeding methods, or compare how different algorithms perform when applied to the same data. The package's vignette provides details and guided examples of how to perform NMF in a variety of common situations. See the link to the package's CRAN web page in section *Availability and requirements*.

### Comparing methods

A typical task in data analysis or algorithm development is to compare how different methods perform on a given data set. We provide a functionality to compare different NMF runs, based on a set of quality measures that have been proposed in the literature to evaluate NMF performance.

Standard measures for evaluating algorithms are the final error between the target matrix and its estimate, or the CPU time required to perform the factorization. Hoyer [12] defined a measure for the *sparseness *of a factorization, which he used as a constraint in the formulation of problem (2). This measure was also used by Pascual-Montano et al. [13], to compare their model to other NMF approaches. They used it in combination with the explained variance, defined as , where the *V*_*ij *_are the entries of the target matrix *V*, and  is the residual sum of squares between the target matrix and its NMF estimate (). This evaluates how well the NMF model reconstructs the original data.

Pascual-Montano et al. studied the deterioration of the explained variance, as a function of sparseness for different methods, to show that their method maintained a good fit for a wide range of achieved sparseness. Note that users should be cautious about using it as the basis for comparing the performance of different methods, since it is closely related to the objective function of methods based on euclidean distance but not for Kullback-Leibler divergence, and would a priori favor the former methods. On the other hand, the results from [13] showed that algorithms not based on the euclidean distance may still achieve better values of explained variance than euclidean-based methods, especially when these include regularization terms. Further work would be required to investigate better the implications of differences in the objective function's underlying metric, with regards to the RSS values.

Kim and Park [23] used the notions of *purity *and *entropy *to evaluate the quality of a clustering, in cases where there is prior knowledge of the classes to which the samples belong. In the context of clustering or classification studies, Brunet et al. [4] proposed to use the *cophenetic correlation coefficient *as a measure of stability of the clusters. Following a consensus clustering approach [28], they computed the consensus matrix, that is, the average connectivity matrix over multiple runs. From a statistical point of view, this gives the empirical probability for each sample pair to be clustered together. Considering the entries of the consensus matrix as similarity measures, the cophenetic correlation coefficient is defined as the correlation between the sample distances induced by the consensus matrix, and the cophenetic distances obtained by its hierarchical clustering [4].

In Table 1 we give an example of the output from the comparison of three NMF methods. The results are for illustrative purposes only and are not intended as a thorough comparison of the relative performance of the methods. Each method was run once, using the non-negative double SVD (NNDSVD) method from [27] to seed the computation. The quality measures are computed for each method and displayed together with some extra characteristics such as the algorithm's name, the rank of factorization, or the metric on which the loss function that estimates the approximation error in (2) is based. In the metric column, the labels "KL" and "euclidean" stand for the Kullback-Leibler divergence and the Frobenius norm respectively. The *evar *column gives the values obtained for the explained variance.

**Table 1:** Comparison of NMF methods

method	seed	metric	rank	evar	sparseness W/H	purity	entropy	niter	CPU time (seconds)
lee	nndsvd	euclidean	3	0.75	0.65/0.75	0.89	0.25	690	11.24
snmf/r	nndsvd	euclidean	3	0.75	0.65/0.75	0.97	0.10	130	4.31
brunet	nndsvd	KL	3	0.73	0.64/0.80	0.95	0.16	1110	23.60
nsNMF	nndsvd	KL	3	0.70	0.73/0.74	0.87	0.29	450	10.37

Comparison of different NMF algorithms applied to the Golub dataset, using the non-negative double SVD seeding method (NNDSVD). The *metric *column provides the metric associated with each method: "euclidean" stands for Frobenius norm, "KL" for Kullback-Leibler divergence.

### Estimating the factorization rank

A critical parameter in NMF is the factorization rank *r*. It defines the number of metagenes used to approximate the target matrix. Given a NMF method and the target matrix, a common way of deciding on *r *is to try different values, compute some quality measure of the results, and choose the best value according to this quality criteria.

The most common approach is to use the cophenetic correlation coefficient. Brunet et al. [4] suggested choosing the smallest value of *r *for which this coefficient starts decreasing. Another approach proposed by Hutchins et al. [6] is based on the variation of the residual sum of squares (RSS) between the target matrix and its estimate. They used Lee and Seung's standard algorithm for Frobenius norm, choosing the optimal factorization rank as the value of *r *for which the plot of the RSS shows an inflection point. Whereas the standard NMF procedure usually involves several hundreds of random initialization, performing 30-50 runs is considered sufficient to get a robust estimate of the factorization rank [4,6].

**Figure 1:**
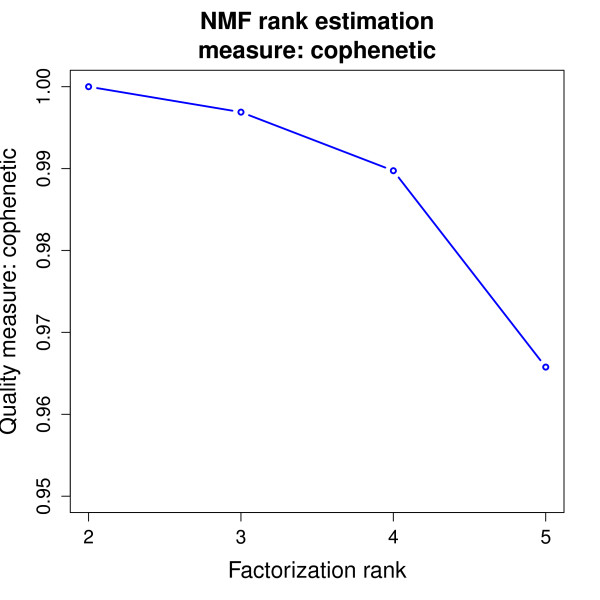
**Cophenetic correlation coefficient**. Each point on the graph was obtained from 50 runs of the Brunet et al's algorithm [4]. This graph indicates the robustness of the clusters for different values of the factorization rank. There is a large decrease in the stability for *r *= 5, compared to lower ranks.

The NMF package implements the above mentioned procedures and provides functions to generate plots for the different quality measures. To illustrate this functionality, we reproduce Brunet et al.'s estimation of the optimal factorization rank. Figure 1 shows the plot of the cophenetic correlation coefficient for *r *in the range 2-5. Each point on the graph was obtained from 50 runs of the Brunet et. al's algorithm [4]. As pointed out in [4], the cophenetic coefficient indicates the robustness of the clusters obtained for a given choice of *r*. For *r *= 2, the clusters obtained after each of the 50 runs are the same, as reflected by a perfect consensus matrix - with only 0 and 1 entries - and a cophenetic correlation coefficient equal to one in Figure 1. Despite a decrease in the cophenetic correlation coefficient for *r *= 3; 4, the clusters are still deemed robust by Brunet et al. [4], who selected the model with *r *= 3, which produces meaningful results that match actual phenotypic classes. They considered that the biological significance of the fourth cluster, for *r *= 4, is less clear. The sharp decrease in the cophenetic correlation coefficient at rank *r *= 5 indicates that substantially less stability is achieved using more than four clusters.

**Figure 2:**
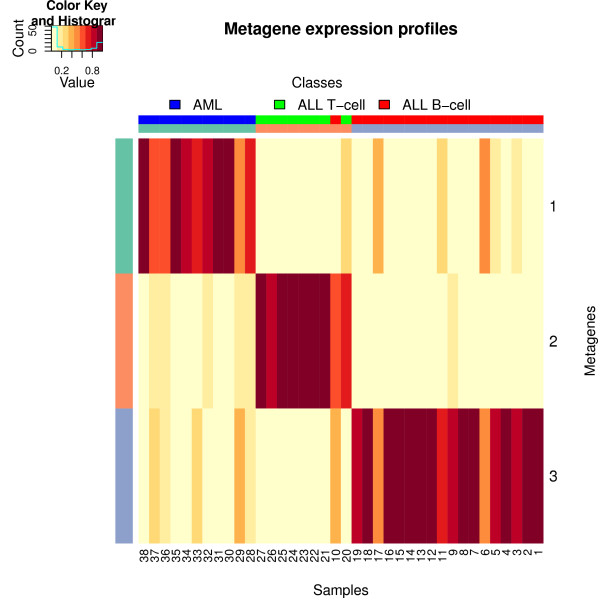
**Heatmap of the metagene expression profiles matrix**. The metagene expression profile matrix was obtained from the factorization that achieved the lowest approximation error across 200 random runs of the Brunet et al.'s algorithm on the Golub dataset. Each column corresponds to a samples. The top colored row shows the phenotypic class to which each sample belongs. Columns were scaled to sum to one and ordered by clusters, which are highlighted on the second row by colours that map them with their associated metagene.

This approach does not always provide a clear and consistent cut-off for the choice of *r *[9]. Frigyesi et al. [29] objected that the cophenetic correlation coefficient evaluates more the ability of each value of the rank to classify the samples into the same number of classes, rather than the actual optimal value of *r*, which could be smaller. Moreover it does not incorporate any correction that would prevent overfitting the data. An approach to overcome these issues consists in integrating the results obtained from the factorization of random data into the estimation of *r *[29,30]. Indeed, an increase in *r *is relevant if the information captured by the factorization is greater than that obtained from random unstructured data, otherwise the increase in *r *is likely to result in overfitting. Frigyesi et al. [29] selected *r *as the smallest factorization rank for which the marginal decrease in the residuals remains larger than the decrease observed for randomized data. The stability of the clustering is assessed in a second step, using the cophenetic correlation coefficient obtained from a modified version of the consensus matrix. The modification consists in weighting the connectivity matrix of each run according to its relative residual error among all the runs [29].

### Computational speed

Performing a single NMF run on large scale data requires intensive computations. Moreover, a typical NMF analysis involves performing several runs for different values of the rank (~ 30-50 runs), before running the final factorization using the estimated rank (~ 200 runs) The whole procedure is therefore highly time consuming.

Since R is able to call external compiled libraries, one possible way to speed-up the computations is to implement optimized versions of the algorithms in C/C++ or Fortran. For instance, the NMF package implements optimized C++ versions of the multiplicative updates from [4,10], which are used by several other NMF algorithms [13,24,31]. On another level, given that the runs are independent one from another, performing the computations in parallel can lead to a significant speed improvement. The R System provides a number of ways to parallelize the execution of code. The NMF package implements multiple runs within the parallel computing framework developed by REvolution Computing [32], which allows parallel computations to be run transparently in multi-core environments. A step further in parallelism, consists in distributing the runs across several machines, using one of the available R packages that provides interfaces to high-performance computing (HPC) cluster environments.

As an example, we provide here the computation times achieved when running 100 factorizations of the 5000 × 38 gene expression matrix from the Golub dataset, using Brunet et al.'s algorithm with *r *= 3. It took 8.5 min to run all the factorizations sequentially, 4.8 min when using multi-core computation alone (see the hardware specification above), and 2.5 min when distributed over 4 quadri-core nodes on a HPC cluster (using Sun Grid Engine [33]). Besides the memory used by the R session itself, a single NMF run of Brunet's algorithm (with *r *= 3) required on average 25Mb. The Golub dataset and the fitted rank-3 factorization used 850Kb and 450Kb respectively.

### Visualizing results

R includes a wide range of powerful plotting utilities. However, producing interpretable plots often requires tuning several function arguments, which can act as a distraction from the main analysis task. To help in interpreting and evaluating the estimated factorization, our package implements a collection of functions pre-configured to visualize the results from NMF runs. Each visualization method provides insights about specific characteristics of the result or the method used.

#### Sparse parts-based representation

**Figure 3:**
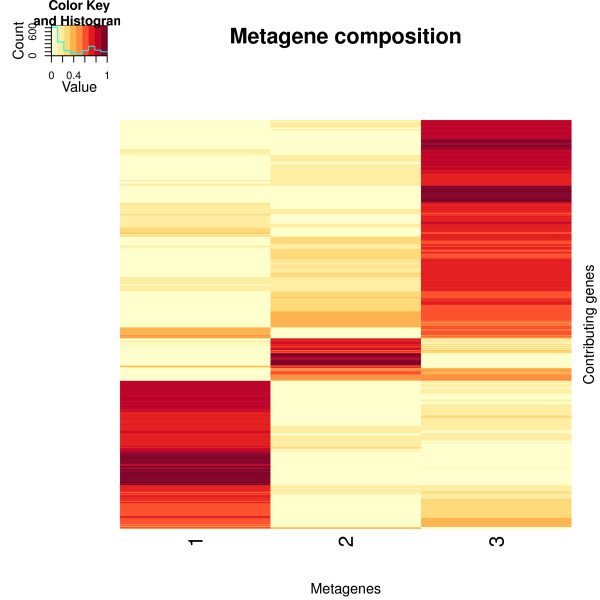
**Heatmap of the metagene matrix**. The metagene matrix was obtained from the same factorization used in Figure 2. Each row corresponds to a gene. The most metagene-specific genes were selected using the Kim and Park's scoring and filtering method. This resulted in the selection of 635 genes. Rows were scaled to sum to one and ordered by hierarchical clustering based on the euclidean distance and average linkage.

One of the main properties of NMF is its ability to produce metagenes or metagene expression profiles that have a sparse structure. This feature is exploited in practice to simultaneously define and characterize clusters of genes and samples [3,8]. A common way to highlight whether any sparse or interesting overlapping patterns have been recovered by the algorithm is to draw heatmaps of both factor matrices. Figures 2 and 3 show the heatmaps generated from the metagene expression profiles (*H*) and metagene matrices (*W*) respectively. Both matrices were obtained from the same factorization, that is the one that achieved the lowest approximation error across 200 random runs of the Brunet et al.'s algorithm. High values are displayed in red, small values in yellow.

In Figure 2, to emphasize the relative contribution of each metagene to each sample, the columns are scaled to sum to one. If prior knowledge about sample classes is available, it can be conveniently added to the plot. Here we used the cancer subtype information that is available for each ALL sample. The first row at the top of the heatmap shows ALL-T samples in green, ALL-B in red, and the AML samples in blue. The samples are clustered based on the metagene that most contributes to their expression profile. The second top row highlights these clusters, and maps them to the corresponding metagene using the same colours as the left side column. This allows each metagene to be associated with the sample class in which it is most highly expressed. In this case, metagene 1 is associated with the AML group, metagene 2 with the ALL-T group, and metagene 3 with the ALL-B group.

The metagene matrix, *W*, contains the contribution of several thousand genes to each metagene. Due to its sparse structure, a heatmap of *W *showing all the genes is usually not easily interpretable. The NMF package implements a scoring and selection method proposed in [23] to extract the most metagene-specific genes. First, the genes are scored based on their contributions to each metagene. Then the subset of genes that score higher than some threshold, s, and for which the maximal contribution in *W *is larger than the median of all elements in *W *are selected. The score threshold *s *is computed from the gene score vector itself as s = *μ *+ 3*σ *, where *μ *and *σ *are respectively the median and the median absolute deviation of the gene scores. Figure 3 shows the 635 genes selected in the case of the Golub dataset. The sparse structure of the metagenes clearly appears in the localized patterns of the gene contributions.

#### Cluster stability

**Figure 4:**
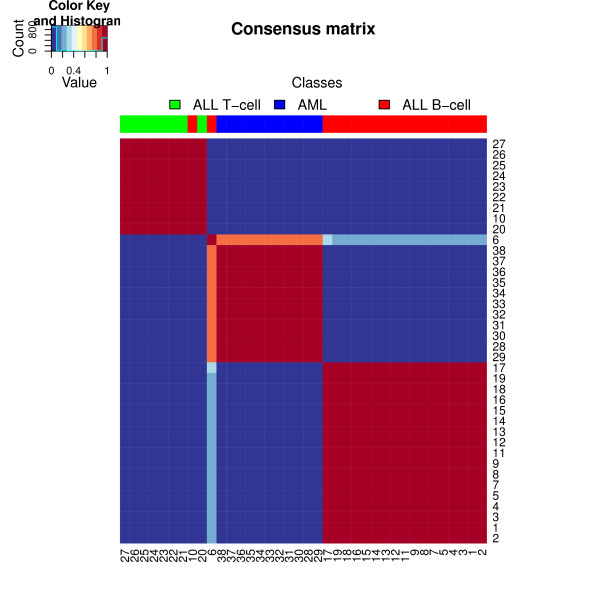
**Consensus matrix**. The consensus matrix was obtained from 200 random runs of the Brunet et al.'s algorithm on the Golub dataset. Values range from 0 to 1. Columns - and rows - were ordered by hierarchical clustering based on the euclidean distance with average linkage.

In the context of sample clustering, the consensus matrix provides information about the stability of the clusters defined by the metagene expression profiles [4,9]. For each cluster, it distinguishes the samples that are difficult to classify from those that consistently cluster together. Figure 4 shows the heatmap of the consensus matrix obtained after 200 random runs of the Brunet et al. algorithm. Zero entries are shown in dark blue and one entries in red. The clear block structure indicates strong stability of the clusters. Except for samples number 10 and 6, all of the samples are clustered consistently across runs, recovering the three cancer classes. Sample 10 (ALL_21302) is an ALL_B sample and is consistently clustered with ALL_T samples. Sample 6 (ALL_14749), another ALL_B sample, appears to be alternatively clustered with the AML samples or the other ALL_B samples. However, the heatmap shows that it more often clusters with the AML subtype. These results are consistent with the observations reported in [4,9].

#### Convergence speed

**Figure 5:**
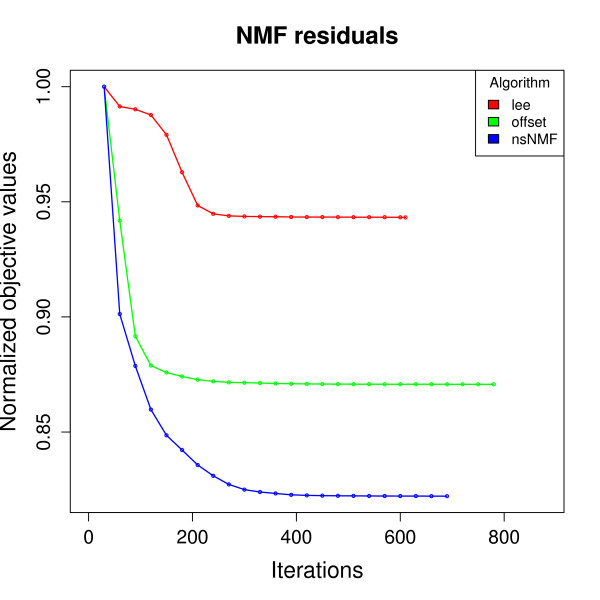
**Plot of the residual approximation error**. Each curve reports the trajectory of the approximation residuals, computed with the algorithm's loss function. Each track is normalized separately over its maximum value, and stops at the number of iterations required to achieve the convergence criterion.

Finally, when developing new algorithms or comparing results, the graph of the residual approximation error provides information about the convergence speed and efficiency of each method. The NMF package provides a built-in functionality to track the objective value along the iterative optimization process. Figure 5 illustrates a residual plot produced from the comparison of three methods, which were run once using a common random starting point. For all algorithms we used the same convergence criterion, which is based on the invariance of the consensus matrix such as defined in [4], and considers that convergence is achieved when the consensus matrix does not change over 40 iterations. All algorithms appear to achieve stationarity of the objective function after a few hundred iterations, but need to run longer to satisfy the convergence criterion. Lee and Seung's algorithm [10] was the first to converge, although achieving the least decrease in the residuals. The NMF with offset algorithm [24] converged somewhat later than the other two.

## Conclusions

Nonnegative Matrix Factorization has several advantages over classical approaches to extracting meaningful information from high-dimensional data. Its successful application in many fields, notably in bioinformatics, has resulted in the development of several algorithms and methodologies. However, the implementations available for these algorithms often depend on commercial software or require technical skills. We implemented the NMF package to provide free and simple access to standard methods to perform Nonnegative Matrix Factorization in R/BioConductor. The package also provides a flexible framework that allows the rapid development, testing and benchmarking of novel NMF algorithms.

## Availability and requirements

**Operating system: **Any

**Dependencies: **R (≥ 2.10)

**Optionally: **BioConductor (≥ 2.5)

**Programming Language: **R, C++

**License: **GPL

**Web: **http://cran.r-project.org/package=NMF

## List of abbreviations used

ALL: Acute Lymphoblastic Leukemia; AML: Acute Myeloid Leukemia; ICA: Independent Component Analysis; NMF: Nonnegative Matrix Factorization; nsNMF: Non-smooth NMF; OS: Operating System; PCA: Principal Component Analysis; RSS: Residual Sum of Squares; SVD: Singular Value Decomposition.

## Authors' contributions

RG designed and implemented the software and drafted the manuscript. CS instigated the study, and participated in its design and coordination. Both authors read and approved the final manuscript.
